# Small vessel microembolization and acute glomerulonephritis following infection of aesthetic filler implants

**DOI:** 10.1186/s13000-016-0453-y

**Published:** 2016-01-08

**Authors:** Pablo Cannata-Ortiz, Carolina Gracia, Youssef Aouad, Antonio Barat, Miguel Angel Martinez-Gonzalez, Gabriela Rossello, Catalina Martin-Cleary, Beatriz Fernández-Fernández, Luis Requena, Alberto Ortiz

**Affiliations:** Pathology, IIS-Fundacion Jimenez Diaz, School of Medicine, UAM, Madrid, Spain; Nephrology and REDINREN, IIS-Fundacion Jimenez Diaz, School of Medicine, UAM, Madrid, Spain; Pathology, 12 Octubre Hospital, Madrid, Spain; Dermatology, IIS-Fundacion Jimenez Diaz, School of Medicine, UAM, Madrid, Spain; IRSIN, Madrid, Spain; Laboratory of Nephrology, IIS-Fundacion Jimenez Diaz, Avda Reyes Catolicos 2, 28040 Madrid, Spain

**Keywords:** Esthetic filler implants, Polymethylmetacrylate, C3 glomerulopathy, Post-infectious glomerulonephritis, Nephritic syndrome

## Abstract

**Background:**

The systemic consequences of esthetic filler injections are poorly understood.

**Case presentation:**

We report a patient with a past history of subcutaneous injection of aesthetic filler material in the lower legs, who presented with post-infectious glomerulonephritis following necrotic leg ulcers at the injection site. Kidney biopsy revealed the presence of translucent, non-birefringent microspherical bodies compatible with polymethylmetacrylate (PMMA) microspheres in some capillary lumens. This had not previously been described. PMMA is a biphasic aesthetical filler composed of polymethylmetacrylate microspheres suspended in a biodegradable bovine collagen carrier. The solid phase (PMMA microspheres) persists in tissues for years. Although PMMA was thought to not disseminate systemically, tissue necrosis may have favored systemic dissemination of the microspheres, although entry in the circulation and microembolization at the time of administration cannot be ruled out.

**Conclusions:**

In conclusion, aesthetic filler implants may cause microembolization into small vessels. Recognition of the characteristic morphology may expedite diagnosis and avoid unnecessary further testing.

## Background

Injected filler agents are used to treat skin wrinkles and for soft tissue augmentation for cosmetic reasons. However, fillers may induce adverse inflammatory reactions that may require biopsy for adequate characterization. Slowly biodegradable or non-resorbable fillers may cause severe reactions that may appear years after the injection, when the patient does not remember which product was injected. Histopathology is the gold standard technique to identify the responsible filler [1]. However, fillers are thought to remain locally at the site of injection and unexperienced pathologists may have trouble identifying the images as aesthetic fillers when found in an internal organ. This adds to the complexity of providing an accurate diagnosis as illustrated by this case of glomerulonephritis following infection of subcutaneous filler material.

## Case presentation

A 57-year-old woman was admitted for painful lower leg ulcers. Past history included subcutaneous injection of esthetic filler material in the lower legs 20 years ago, hypothyroidism and HIV infection on highly active antiretroviral therapy (HAART: raltegravir, abacavir, lamivudine). Viral load was negative and CD4 cells were 600/μl. One year before admission, bilateral torpid lower leg ulcers developed after minor trauma and progressively expanded. She was treated with diclofenac.

On admission blood pressure was 190/88 mmHg (previously normal) and she had extensive necrotic ulceration and edema in both lower legs. Key lab results were serum creatinine 1.5 mg/dl (previously 1.0 mg/dl), serum albumin 3.5 mg/dl, serum cholesterol 139 mg/dl, complement factor 3 (C3) nadir 63 mg/dl, normal C4, polyclonal hypergammaglobulinemia, urinary albumin:creatinine ratio 2082 mg/g, urinary protein:creatinine ratio 3557 mg/g and urinary sediment containing 20–40 red blood cells/high power field. ANCA, ANA, and anti-GBM were negative. Ulcer cultures grew Group A streptococcus pyogenes.

**Fig. 1:**
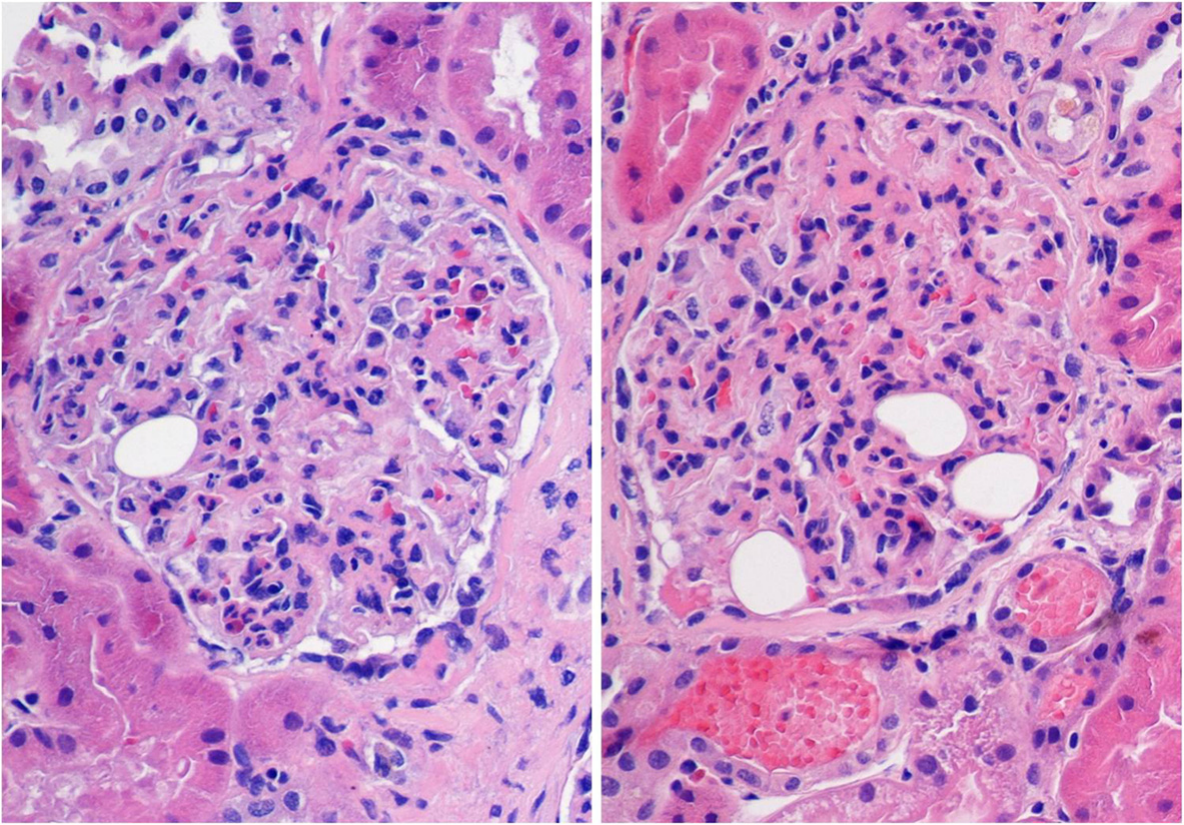
Diffuse proliferative glomerulonephritis with leukocytic infiltration and polymethyl methacrylate microspheres in glomerular capillary lumens (HE ×400)

**Fig. 2:**
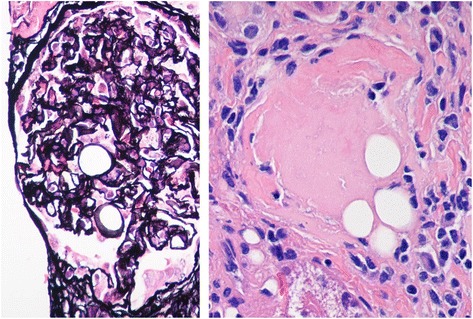
**a** PMMA microspheres in a glomerulus (Jones silver stain × 400). **b** The finding of PMMA in a sclerosed glomerulus supports the permanent nature of this aesthetic filler (HE ×400)

**Fig. 3:**
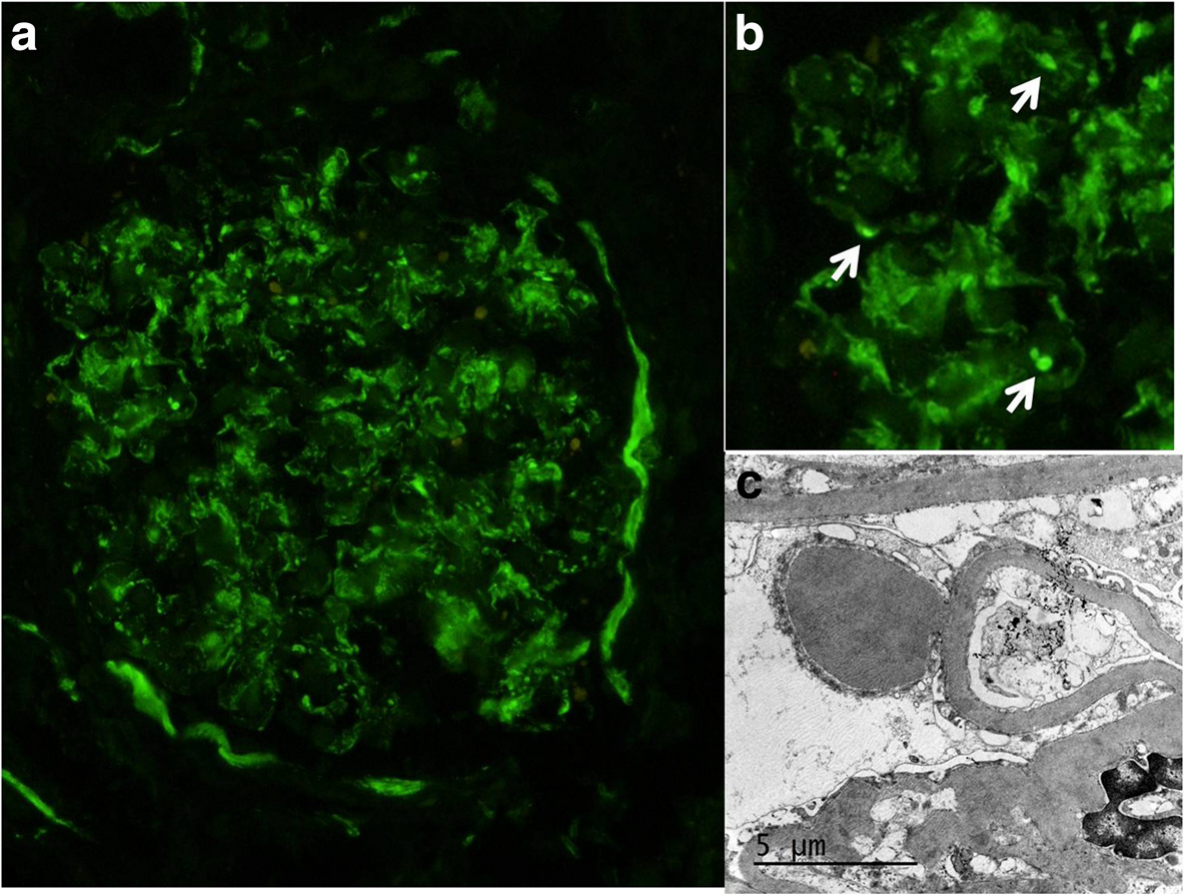
**a** Immunofluorescence showing coarse granular C3 deposition along mesangial region with focal peripheral extension adopting a ‘garland’ pattern. **b** A detail of the glomerular tuft segment between 9 and 11 o’clock. Arrowheads pointing subepithelial deposits that correspond to the hump-shaped deposits seen by EM (box **c**). (**a**: IF anti-C3 ×200; **c**: TEM ×20000)

Kidney biopsy (two cores; 9 and 11 mm long) containing 26 glomeruli, 2 of them completely sclerosed, disclosed diffuse proliferative glomerulonephritis characterized by glomerular hypercellularity due to mesangial proliferation and endocapillary leukocytic infiltration (Fig. 1). Trichrome staining revealed subepithelial fuchsinophilic deposits. Jones silver stain did not show spikes or basement membrane reduplication (Fig. 2). Coarse mesangial and peripheral capillary loop staining for C3 was seen on immunofluorescence (Fig. 3), without IgG, IgA or IgM deposition. Round translucent, non-birefringent microspherical bodies (30–35 μm) resembling adipocytes were identified within some capillary lumens (Figs. 1 and 2). Microspheres were also seen in a globally sclerosed glomerulus (Fig. 2). In the tubulointerstitial compartment, intratubular red blood cell casts, acute tubular damage and patchy interstitial lymphocytic infiltrates were noted. Vessels showed arteriolar hyalinosis and moderate myointimal hyperplasia. Ultrastructural study revealed subepithelial deposits (‘humps’) (Fig. 3).

**Fig. 4:**
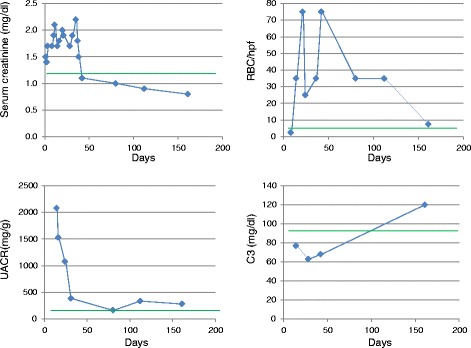
Time-course of changes in serum creatinine (mg/dl), urinary albumin/creatinine ratio (UACR in mg/g), hematuria expressed as red blood cells per high power fields (RBC/hpf) and serum complement factor 3 (C3, mg/dl). Green line denotes normal values limit

Treatment included local debridement, antibiotics and angiotensin converting enzyme inhibitors. Estimated glomerular filtration rate, C3 and microhematuria normalized at month 3, 5 and 12, respectively, while albuminuria progressively improved over the next 12 months (Fig. 4). One year after the initial admission, angiotensin converting enzyme inhibitors had been stopped and serum creatinine was 0.9 mg/dl, estimated glomerular filtration rate 94 ml/min/1.73 m2, urinary albumin:creatinine ratio 188 mg/g and urinary sediment contained no red blood cells/high power field.

## Discussion

The initial differential diagnosis included acute kidney injury due to nonsteroidal anti-inflammatory agents, acute or rapidly progressive glomerulonephritis that may have been superimposed on prior antiretroviral nephrotoxicity or chronic HIV-associated glomerulonephritis [2]. Serological studies and renal biopsy narrowed the differential diagnosis to acute postinfectious glomerulonephritis or C3 glomerulopathy, while a potential role of the implanted cosmetic filler material remained unclear.

Morphology of glomerulonephritis with dominant C3 deposits may underlie both post-infectious glomerulonephritis and C3 glomerulopathy, a recently introduced pathological entity [3]. Currently, C3 glomerulopathy is defined as a disease process due to abnormal control of complement activation, deposition, or degradation and characterized by predominant glomerular C3 deposition with electron-dense deposits. However, true post-infectious glomerulonephritis cannot often be differenced from C3 glomerulonephritis based on morphological, clinical and laboratory data available at the time of biopsy. Rather, follow-up of clinical parameters, serum C3 levels and urinary abnormalities over several months may differentiate both entities [2]. As in the present case, the decreased serum C3 levels will normalize over 8–12 weeks in post-infectious glomerulonephritis, although hematuria and albuminuria may persist for years [4]. The presence of Group A streptococcus pyogenes infection, the time course of kidney and immunological abnormalities, the renal biopsy and the absence of evidence of abnormal control of complement activation, deposition, or degradation, all support a diagnosis of post-infectious (post-streptococcal) glomerulonephritis. These features also argue against HIV associated glomerulonephritis. The patient was not taking HAART drugs that are clearly nephrotoxic, such as tenofovir, indinavir or atazanavir [5–7]. In this regard, no evidence of proximal tubulopathy (tenofovir) or characteristic urine or kidney tissue crystals (indinavir, atazanavir) were observed and toxic effects of hematuria may account for tubular injury features observed in the biopsy [8].

The presence of microspheres in the kidney biopsy was striking. While the patient did not recall the nature of the injected material, the histologic appearance was consistent with polymethylmetacrylate (PMMA), a biphasic aesthetical filler composed of biodegradable bovine collagen and PMMA microspheres which persist in tissues for years [1]. There is longstanding clinical experience with this material and only local (subcutaneous) inflammatory granulomatous reactions are documented [9]. We are not aware of prior reports of systemic dissemination of PMMA microspheres in humans. PMMA was thought to not disseminate systemically, but it may generate local inflammatory reactions spontaneously or following a trigger [10, 11]. The injection of the filler several years ago may explain the absence of a local kidney inflammatory granulomatous reaction to PMMA microspheres, and their presence in a sclerosed glomerulus supports their permanent nature. The presence of PMMA in the lower legs may have favored the torpid course of the ulcers. Conversely, tissue necrosis may have favored systemic dissemination of the microspheres, although we can only speculate as to their contribution to the clinical presentation. PMMA does not appear to have triggered the present inflammatory response since emboli were present in the absence of local inflammation. However, it may have contributed to local ischemia and tubular injury. Entry in the circulation and microembolization at the time of filler administration, many years ago, cannot be ruled out.

## Conclusions

The final diagnosis was acute post-streptococcal glomerulonephritis with esthetic filler implants microembolization into small vessels. Esthetic filler implant microembolization is here described for the first time. We conclude that esthetic filler implants may cause microembolization into small vessels. Recognition of the characteristic morphology may expedite diagnosis and avoid unnecessary further testing.

## Consent

Written informed consent was obtained from the patient for publication of this Case Report and any accompanying images. A copy of the written consent is available for review by the Editor-in-Chief of this journal.
